# Palmitate-Triggered COX2/PGE2-Related Hyperinflammation in Dual-Stressed PdL Fibroblasts Is Mediated by Repressive H3K27 Trimethylation

**DOI:** 10.3390/cells11060955

**Published:** 2022-03-10

**Authors:** Lisa Schuldt, Michael Reimann, Katrin von Brandenstein, Julia Steinmetz, Annika Döding, Ulrike Schulze-Späte, Collin Jacobs, Judit Symmank

**Affiliations:** 1 Orthodontic Research Laboratory, Department of Orthodontics, University Hospital Jena, Leutragraben 3, 07743 Jena, Germany; lisa.schuldt@krz.uni-jena.de (L.S.); katrin.brandenstein@med.uni-jena.de (K.v.B.); julia.steinmetz@uni-jena.de (J.S.); 2 Section of Geriodontics, Department of Conservative Dentistry and Periodontics, University Hospital Jena, Leutragraben 3, 07743 Jena, Germany; michael.reimann@med.uni-jena.de (M.R.); annika.doeding@med.uni-jena.de (A.D.); ulrike.schulze-spaete@med.uni-jena.de (U.S.-S.); 3 Center for Dental, Oral and Maxillofacial Medicine, Department of Orthodontics, University Hospital Jena, 07743 Jena, Germany; collin.jacobs@med.uni-jena.de

**Keywords:** periodontitis, tooth movement, obesity, palmitic acid, histone modification, inflammation, COX2/PGE2, IL-10

## Abstract

The interrelationships between periodontal disease, obesity-related hyperlipidemia and mechanical forces and their modulating effects on the epigenetic profile of periodontal ligament (PdL) cells are assumed to be remarkably complex. The PdL serves as a connective tissue between teeth and alveolar bone and is involved in pathogen defense and the inflammatory responses to mechanical stimuli occurring during tooth movement. Altered inflammatory signaling could promote root resorption and tooth loss. Hyperinflammatory COX2/PGE2 signaling was reported for human PdL fibroblasts (HPdLFs) concomitantly stressed with *Porphyromonas gingivalis* lipopolysaccharides and compressive force after exposure to palmitic acid (PA). The aim of this study was to investigate the extent to which this was modulated by global and gene-specific changes in histone modifications. The expression of key epigenetic players and global H3Kac and H3K27me3 levels were quantitatively evaluated in dual-stressed HPdLFs exposed to PA, revealing a minor force-related reduction in repressive H3K27me3. UNC1999-induced H3K27me3 inhibition reversed the hyperinflammatory responses of dual-stressed PA cultures characterized by increased *COX2* expression, PGE2 secretion and THP1 adhesion. The reduced expression of the gene encoding the anti-inflammatory cytokine IL-10 and the increased presence of H3K27me3 at its promoter-associated sites were reversed by inhibitor treatment. Thus, the data highlight an important epigenetic interplay between the different stimuli to which the PdL is exposed.

## 1. Introduction

Genetic predisposition, unfavorable environmental conditions and an unhealthy lifestyle are important risk factors for the onset and progression of periodontal disease, but they cannot fully account for individual susceptibility [1]. In light of the high prevalence of this chronic, non-communicable disease and its subsequent economic and healthcare implications [2], the investigation of additional key regulatory mechanisms has been the focus of recent periodontitis research. This has drawn attention to epigenetic regulatory mechanisms as important factors in the pathogenesis of the disease [3]. Epigenetic modifications include, in part, heritable histone patterns, which restrict the accessibility of DNA for the transcription machinery without altering the genome [4]. With regard to the inflammatory aspects of periodontal disease, post-translational modifications (PTMs) to histone tail amino acids have been thoroughly investigated [5]. PTMs to histones include acetylation and methylation, among others, which modulate transcriptional activity in a context-dependent manner [6]. While the attachment of acetyl groups by histone acetyltransferases (HATs) is generally associated with an opening of chromatin structure and a beneficial environment for gene expression, the effects of histone methylation depend on the amino acids being modified and the abundance of these methyl groups [6,7]. For example, the trimethylation of lysine at position 27 (K27) on histone 3 (H3) has been linked to transcriptional repression [7]. Regarding the expression of key factors regulating PTMs, a number of comparative studies have shown differences between periodontal patients and healthy subjects [5], presumably caused by the pathogenic infections and possibly linked to excesses of bacterial metabolites, such as short-chain fatty acids [8].

Elevated serum levels of long-chain fatty acids, such as the saturated fatty acid palmitic acid (PA), are typically associated with obesity and have been investigated for their role in disease-related low-grade systemic inflammation [9,10,11]. Furthermore, a pro-inflammatory characteristic of PA has been shown under hyperlipidemic conditions [12,13,14,15,16,17,18,19,20], although, in general, fatty acids are involved in normal cell functions [21]. Several studies have reported an interrelationship between both diseases. For example, a delayed response to an infection with the Gram-negative oral anaerobe *Porphyromonas gingivalis* (*P. gingivalis*) was observed in mice fed with a high-fat diet [22]. *P. gingivalis* has been described as a keystone pathogen affecting oral health and disease, possibly due to its unique ability to evade the host immune response [23]. Although various changes in the host oral environment and bacterial biofilm composition are required for the onset and progression of periodontitis, the pro-inflammatory effect of *P. gingivalis’* lipopolysaccharides (LPS) is commonly used to mimic periodontitis-causing conditions in vitro.

Even though inflammatory processes are increased in both diseases, their concomitant impact on the inflammatory response of the periodontal ligament (PdL) when subject to mechanical forces, such as those exerted during trauma, mastication or orthodontic tooth movement, remains poorly studied. The PdL is the connective tissue between the teeth and alveolar bone, and its most abundant cells, PdL fibroblasts (PdLFs), modulate the transient, aseptic inflammatory response to compressive forces in a temporal and spatial manner [24,25,26].

Alterations in the inflammatory response of PdLFs may increase the risks of tooth root resorption and tooth loss, primarily by affecting tissue- and bone-remodeling cells [27,28]. In rats suffering from periodontitis, the upregulated expression of several cytokines in the PdL and the enhanced presence of activated osteoclasts were observed after tooth movement, in addition to increased dorsal root resorption [29]. In light of the tremendous heterogeneity of obesity- and hyperlipidemia-associated cellular adaptions, in vivo studies can be quite challenging to conduct. Thus, even the results of prospective controlled clinical trials only examining the effects of increased BMI on orthodontic tooth movement (OTM) are conflicting [30,31,32,33].

However, influences on epigenetic regulatory mechanisms are evident with regard to high-fat diets and obesity-associated alterations [34]. Relatedly, increased histone acetylation was reported in several cell types under hyperlipidemic conditions, potentially due to the role of fatty acids as lipid-based alternative donors for acetyl-CoA, which is required for this epigenetic mark [21,35]. In addition, a potential influence of PA on repressive methylated histone marks has recently been reported [36,37]. This could provide a framework for understanding the interplay among periodontitis-induced changes and a target for potential therapeutic interventions [38].

We recently reported that PA-stimulated human PdL fibroblasts (HPdLFs) showed an excessive inflammatory response to simultaneous stimulation with *P. gingivalis* LPS and compressive force, mainly through the enhanced secretion of prostaglandin E2 (PGE2) [39], which is regulated by cyclooxygenase 2 (COX2) [40]. The aim of this study was to investigate whether this excessive inflammation was mediated by epigenetic alterations induced by palmitic acid.

## 2. Materials and Methods

### 2.1. Cell Culture

Culture medium consisting of Dulbecco’s modified Eagle medium (DMEM; Thermo Fisher Scientific, Carlsbad, CA, USA), 4.5 g/L glucose, 10% heat-inactivated fetal bovine serum (Thermo Fisher Scientific), 100 U/mL penicillin, 100 μg/mL streptomycin and 50 mg/L L-ascorbic acid was used for growing commercially acquired human periodontal ligament fibroblasts (HPdLFs, Lonza, Basel, Switzerland) at 37 °C, under 5% CO_2_ and 95% humidity. The HPdLFs were passaged at a confluency of 75% with 0.05% Trypsin/EDTA (Thermo Fisher Scientific) and used for experiments at passages four to eight.

RPMI 1640 medium (Thermo Fisher Scientific) containing 10% FBS, 100 U/mL penicillin and 100 µg/mL streptomycin was used for culturing THP1 cells (DMSZ, Braunschweig, Germany) at 37 °C, under 5% CO_2_ and 95% humidity. Weekly passages were performed, and 1 × 10^6^ cells were seeded into T175 culture flasks for further culture.

### 2.2. Stimulation with Palmitic Acid

For the analysis of RNA expression, 2.5 × 10^4^ HPdLFs were seeded into each well of a 6-well plate. For immunofluorescence staining, 5 × 10^3^ cells were plated onto coverslips in each well of a 48-well plate. The cells were cultured for 24 h in DMEM culture medium prior to six-day stimulation with 200 µM palmitic acid (PA). The PA-containing medium was prepared as previously described [39]. Briefly, dried PA was dissolved at 70 °C in sterile water containing 50 mM NaOH, complexed with 37 °C-preheated bovine serum albumin (BSA, Seqens IVD, Limoges, France) and added to DMEM culture medium. BSA-containing medium was used for control stimulation.

### 2.3. Stimulation with P. gingivalis Lipopolysaccharides

To simulate pathogenic stimulation, 10 µg/mL of lipopolysaccharides (LPS) of *Porphyromonas gingivalis* (*P. gingivalis*; InvivoGen, San Diego, CA, USA) was applied to the culture medium for 6 h in tandem with the application of compressive force.

### 2.4. UNC1999 Application

For the inhibition of H3K27 trimethylation, different concentrations of the EZH1/EZH2 inhibitor UNC1999 (0.25, 0.5 and 1.0 µM) were applied for six hours on 75%-confluent HPdLFs. For further treatment, 1.0 µM UNC1999 was applied for 6 h in tandem with *P. ginigivalis* LPS and the application of a compressive force.

### 2.5. Application of Compressive Force

A compressive force of 2 g/cm^2^ was applied according to the protocol of Kirschneck et al. [41] and as previously reported [42]. Briefly, in tandem with LPS applications, glass plates were placed on fatty acid-cultured HPdLFs for 6 h at 37 °C, under 5% CO_2_ and 95% humidity. Afterwards, the cells were either directly processed using TRIzol^TM^ Reagent (Thermo Fisher Scientific) for expression analysis or isolated using Dulbecco’s Phosphate-Buffered Solution (DPBS, Thermo Fisher Scientific) for histone extraction and, subsequently, protein analysis. In 48-well plates, the compressive force was applied via six-hour centrifugation at 30 °C with a force of 7.13 g/cm^2^. Control cells were cultured at 30 °C for the duration of force application.

### 2.6. Expression Analysis with Quantitative PCR

RNA isolation, cDNA synthesis and quantitative PCR were performed as previously described [43]. The primer sequences used for target amplification are shown in Table 1. The primer quality, specificity and efficiency were analyzed as previously described [43]. *RPL22* and *TBP* were used as reference genes for calculating the relative normalized expression levels. The efficiency (E)-corrected E^-ΔΔCT^ method was used for data analysis [44]. Each condition was tested with at least biological quadruplicates and technical duplicates for each sample.

### 2.7. Immunofluorescent Staining

To detect trimethylated H3K27 after specific treatments, coverslips with cultured HPdLFs were fixed in 4% PFA for 10 min, washed with phosphate-buffered saline (PBS) and incubated with the primary antibody for 1.5 h and the secondary antibody for 45 min. DAPI (Thermo Fisher Scientific; 1:10,000 in PBS) was used for nucleus staining. The following antibodies were used: rabbit anti-human H3K9/14/18/23/27 (ab47915; Abcam, Cambridge, UK; 1:500), mouse anti-human H3K27me3 (ab6002; Abcam; 1:250), goat anti-rabbit Cy5 and goat anti-mouse Cy5 (111-175-144 and 115-175-146; Jackson ImmunoResearch, West Grove, PA, USA; 1:1000).

### 2.8. THP1 Adhesion Assay

To detect pro-inflammatory cytokine secretion by stimulated HPdLFs, a THP1 adhesion assay was performed as previously described [43]. Briefly, non-adhesive THP1 cells were stained with 15 µM Celltracker CMFDA (Thermo Fisher Scientific) for 30 min at 37 °C. After centrifugation for 5 min at 1000× *g*, THP1 cells were washed in RPMI culture medium and 25 × 10^3^ cells were added to the cultured and stressed HPdLFs in each well of a 48-well plate. After cell adhesion for 30 min under pro-inflammatory stimuli, non-activated THP1 cells were carefully washed away with prewarmed PBS. The HPdLFs and adhesive THP1 cells were fixed with 4% PFA for 10 min prior to the staining of the cell nuclei with DAPI (Thermo Fisher Scientific; 1:10,000 in PBS). The numbers of adhesive CMFDA-labeled THP1 cells were determined and are reported in relation to the total numbers of HPdLFs counted via DAPI staining.

### 2.9. MTT Assay

The cell metabolic activity was analyzed using the plate reader Infinite ^®^ M Nano (TECAN, Männedorf, Swiss) with the MTT colorimetric assay (Sigma Aldrich, St. Louis, Missouri, MO, USA) according to the manufacturer’s protocol. The measured OD values were normalized to those for the respective control condition and are reported as relative metabolic activity values.

### 2.10. Nuclear Extraction and Histone Methyltransferase Activity Assay

An EpiQuik Nuclear Extraction Kit (EpiGentek, Farmingdale, New York, NY, USA) was used for nuclear extraction. The activities of H3K27-specific histone methyltransferases were analyzed with an EpiQuik Histone Methyltransferase Activity/Inhibition Assay Kit H3K27 (EpiGentek) according to the manufacturer’s guidelines, with the plate reader Infinite ^®^ M Nano (TECAN).

### 2.11. TUNEL Assay

Apoptotic cells were detected using the ApopTag^®^ Fluorescein In Situ Apoptosis Detection Kit (Sigma Aldrich) on HPdLFs grown on coverslips according to the manufacturer’s protocol.

### 2.12. ELISA

The medium supernatant of the stimulated HPdLFs was isolated 24 h after the application of compressive force to ensure proper protein secretion. To analyze PGE2 secretion, prostaglandin E2 ELISA (PGE2; R&D Systems, Minneapolis, MN, USA) was used according to the manufacturer’s guidelines.

### 2.13. Chromatin-Immunoprecipitation

To analyze the associations of acetylated H3K at the *IL10* gene regions, chromatin immunoprecipitation (ChIP) was performed. First, DNA and protein were crosslinked with 1% formaldehyde in PBS for 10 min, which was then neutralized with 120 mM glycine. Harvested cells were pelleted via centrifugation for 5 min at 1000× *g* at 4 °C. ChIP was performed on batches of 1 × 10^6^ cells with the Zymo-Spin ChIP Kit (Zymo Research, Freiburg, Germany) according to the manufacturer’s protocol with the ChIP-validated antibodies mouse anti-human H327me3 (ab6002; Abcam) and rabbit anti-IgG (ab171870; Abcam). Throughout the ChIP process, one percent of the input was stored for later normalization. The amount of specific DNA fragments bound to H3K27me3 was determined as previously described [45]. Briefly, primer-specific pre-amplification was performed prior to quantitative analysis with the qTOWER3 (Analytik Jena, Jena, Germany) according to the manufacturer’s protocol using the Luminaris Color HiGreen qPCR Master Mix (Thermo Fisher Scientific). The primer sequences used for analysis are displayed in Table 2. The percentage input method was used to normalize the DNA content [46]. The IgG controls were subtracted from the H3K27me3-specific samples.

### 2.14. Microscopy and Image Analysis

The inverted confocal laser scanning microscope TCS SP5 (Leica, Wetzlar, Germany) was used for image acquisition. Fiji software (https://imagej.net/Fiji, accessed on 1 April 2017) was used to analyze the THP1 cell numbers and TUNEL-positive cells and fluorescence intensity of immunostaining. Intensity measurements were performed as previously reported [47]. Briefly, mean gray values (MGVs) were measured for H3Kac/K27me3 staining in the nuclei of 270 cells per condition. Background-subtracted MGVs were visualized as thermal LUT. Adobe Photoshop CS5 (https://adobe.com, accessed on 1 February 2013) was used to produce the figure illustrations. Each TUNEL assay is displayed in pseudo-color (white). Unless otherwise stated, all the experiments were independently repeated at least three times and performed in technical duplicates.

### 2.15. Statistics

The diagrams show the means ± SEs. GraphPad Prism (https://www.graphpad.com, accessed on 1 February 2021) was used for statistical analysis. Significant differences between the different groups were revealed using a one-way ANOVA followed by a post hoc test (Tukey). Significance levels: */#/§ *p*-value < 0.05, **/##/§§ *p*-value < 0.01, ***/###/§§§ *p*-value < 0.001.

## 3. Results

### 3.1. Force-Induced Increase in H3 Lysine Acetylation in Human Periodontal Fibroblasts Is Not Affected by Palmitic Acid

**Figure 1:**
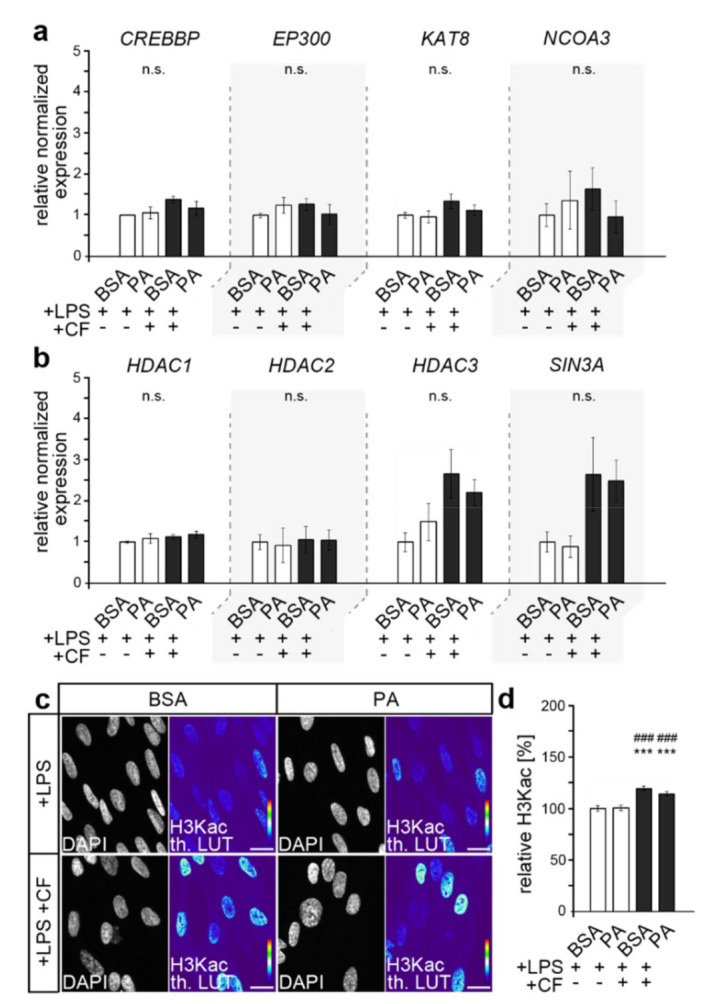
Palmitic acid did not impact H3Kac in compressed HPdLFs stimulated with *P. gingivalis* LPS. (**a**,**b**) Quantitative expression analysis of genes encoding histone acetyl transferases (*CREBBP*, *EP300*, *KAT8*, *NCOA3*) in (**a**) and genes encoding proteins relevant for histone deacetylation (*HDAC1*, *HDAC2*, *HDAC3*, *SIN3A*) in (**b**) in human periodontal ligament fibroblasts (HPdLFs) exposed to palmitic acid (PA) and simultaneously stimulated with compressive force (CF) and *P. gingivalis* LPS and compared to BSA+LPS controls. (**c**,**d**) Representative micrographs of global H3K9/14/18/23/27 acetylation (H3Kac) of HPdLFs under previous conditions. H3Kac staining intensity is shown in thermal LUT (th. LUT) analyzed in relation to BSA+LPS control (**c**). Cell nuclei were visualized with DAPI. *** *p* < 0.001 in relation to BSA+LPS; ### *p* < 0.001 in relation to PA+LPS; n.s., no significant difference between the conditions. One-way ANOVA and post hoc test (Tukey) were used for analysis. Scale bars: 10 μm in (**c**).

Free fatty acids can serve as an alternative source of acetyl-CoA and, thus, affect the H3 lysine acetylation in HPdLFs, as recently reported for oleic acid exposure [48]. To elucidate the role of H3 lysine acetylation (H3Kac) in the regulation of the hyperinflammatory responses of PA-exposed HPdLFs simultaneously stimulated by mechanical and bacterial-induced stress, we first examined the expression of relevant H3Kac regulators (Figure 1a,b), which are associated with PdL properties, periodontal disease and hyperlipidemia [48,49,50,51,52]. These include genes encoding the histone acetyltransferases (HATs) CREB-binding protein (CBP; gene: *CREBBP*), E1A-binding protein p300 (p300; gene: *EP300*), lysine acetyltransferase 8 (KAT8) and nuclear receptor coactivator 3 (NCOA3) and genes encoding proteins relevant for the histone deacetylation, such as histone deacetylase 1 (HDAC1), HDAC2, HDAC3 and proteins that are important for HDAC activity, such as SIN3 transcription regulator family member A (SIN3A). 

Quantitative expression analysis, however, revealed no biologically relevant significant differences under PA treatment, either in genes encoding HATs (Figure 1a) or HDACs or SIN3A (Figure 1b).

However, RNA expression does not necessarily correlate with protein levels, and post-translational regulation may also affect protein activity [53]. For these reasons, we analyzed the level of H3 lysine acetylation through the immunofluorescent pan-staining of H3K9/14/18/23/27ac (H3Kac; Figure 1c,d). We detected increased levels of global H3K acetylation upon the application of compressive forces, supporting recent findings [48]. However, similarly to the expression analysis, no differences were observed due to PA exposure, indicating a rather minor influence of PA for H3Kac under these conditions.

### 3.2. Palmitic Acid Impacts Force-Induced Reduction in H3K27me3 in LPS-Stimulated HPdLFs

Considering that PA can also affect repressive forms of histone methylation, such as H3K27 trimethylation (H3K27me3) [36,37], we then investigated whether alterations in this modification could contribute to triggering the excessive inflammatory stress response. We also performed quantitative PCR for genes encoding core components of the polycomb repressive complex 2 (PRC2), which was already reported to be important for PdL function [54,55]. These include histone methyltransferases Enhancer of Zeste 1 Polycomb Repressive Complex 2 Subunit (EZH1) and EZH2, and SUZ12 Polycomb Repressive Complex 2 Subunit (SUZ12) and Embryonic Ectoderm Development (EED).

**Figure 2:**
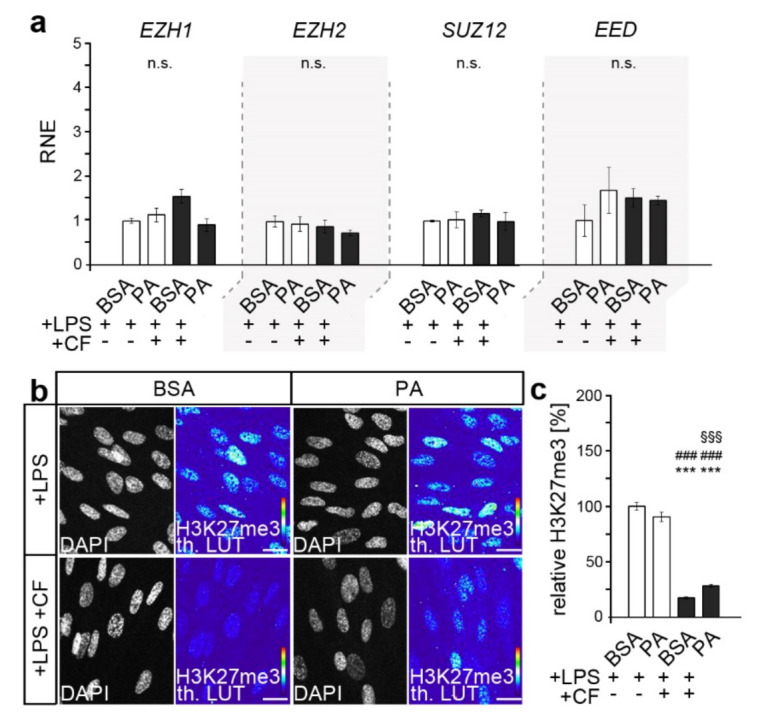
K27 trimethylation in response to dual stimulation was minor in PA-exposed HPdLFs. (**a**) Quantitative expression analysis of genes encoding components of the polycomb repressive complex (*EZH1*, *EZH2*, *SUZ12* and *EED*) in human periodontal ligament fibroblasts (HPdLFs) exposed to palmitic acid (PA) and simultaneously stimulated with compressive force (CF) and *P. gingivalis* LPS and compared to the BSA+LPS controls. (**b**,**c**) Representative microphotographs of global H3K27me3 of HPdLFs under previous conditions. H3K27me3 staining intensity is shown as thermal LUT (th. LUT) in (**c**) in relation to BSA+LPS control. DAPI indicates the nuclei. *** *p* < 0.001 in relation to BSA+LPS; ### *p* < 0.001 in relation to PA+LPS; §§§ *p* < 0.001 in relation to BSA+LPS+CF; n.s., no significant difference between the conditions. One-way ANOVA and post hoc test (Tukey) were used for analysis. Scale bars: 10 μm in (**b**).

As shown in Figure 2a, no relevant changes in gene expression were detected, either due to compressive force or due to fatty acid exposure. However, PRC2′s activity and specificity were shown to be regulated by several post-translational modifications in core components [56]. Therefore, we analyzed the level of H3K27 trimethylation in dual-stimulated HPdLFs with quantitative immunofluorescence (Figure 2b,c).

While the baseline levels were comparable, the application of an additional compressive force led to reduced H3K27me3 levels. However, compared to an 82.68% ± 3.66 reduction in the BSA controls, the changes in the PA cultures (62.17% ± 4.24) were significantly lower (*p*-value 0.0276 × 10^−2^, ***).

### 3.3. Inhibition of PRC2 Enzymes EZH1 and EZH2 Abrogates PA-Induced Excessive Inflammation, Possibly via COX2/PGE2 Modulation

**Figure 3:**
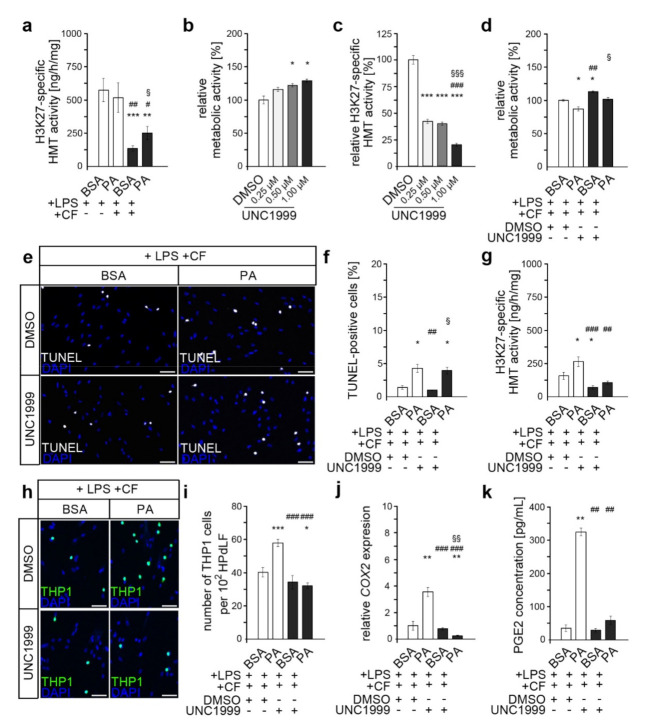
Inhibition of H3K27 trimethylation abrogates the PA-induced excessive inflammatory response of dual-stimulated HPdLFs. (**a**) Analysis of the activity of histone methyltransferases (HMTs) specifically methylating H3K27 in HPdLFs treated with palmitic acid (PA) for six days prior to treatment with *P. gingivalis* LPS and compressive force (CF) for six hours. (**b**) Metabolic activity analyzed in HPdLFs treated with different concentrations of UNC1999 for six hours normalized to DMSO control. (**c**) Analysis of H3K27-specific HMT activity in HPdLFs treated with 1.00 µM UNC1999 in relation to DMSO control. (**d**) Metabolic activity in dual-stressed HPdLFs treated with 1.00 µM UNC1999 in relation to DMSO-treated BSA+LPS+CF control. (**e**) Representative images of TUNEL-positive dual-stressed HPdLFs (white) treated with 1.00 µM UNC1999, with (**f**) indicating the number of apoptotic cells analyzed. (**g**) H3K27-specific HMT activity measured in dual-stressed PA cultures treated with UNC1999. (**h**) Microphotographs of adherent THP1 monocytic cells (green) on compressed and *P. gingivalis* LPS-stimulated HPdLFs after treatment with 1.00 µM UNC1999 in relation to DMSO-treated controls. Cell nuclei were labeled with DAPI (blue). The number of THP1 cells per 10^2^ HPdLFs is displayed in (**i**). (**j**,**k**) Analysis of *COX2* expression levels (**j**) and PGE2 secretion (**k**) in dual-stimulated PA cultures after treatment with 1.00 µM UNC1999 in relation to DMSO-treated BSA+LPS+CF controls. */#/§ *p* < 0.05; **/##/§§ *p* < 0.01; ***/###/§§§ *p* < 0.001; */**/*** in relation to BSA+LPS (**a**), DMSO (**b**,**c**) and BSA+LPS+CF+DMSO (**d**,**f**,**g**,**i**–**k**); #/##/### in relation to PA+LPS (**a**), 0.25 µM UNC1999 (**c**) and PA+LPS+CF+DMSO (**d**,**f**,**g**,**i**–**k**); §/§§/§§§ in relation to BSA+LPS+CF (**a**), 0.5 µM UNC1999 (**c**) and BSA+LPS+CF+UNC1999 (**d**,**f**,**j**). One-way ANOVA and post hoc test (Tukey) were used for analysis. Scale bars: 10 μm in (**h**) and 20 µm in (**e**).

Whether changes in the activity of corresponding H3K27-specific histone methyltransferases (HMTs) could have been responsible for the altered H3K27me3 levels in dual-stressed PA cultures was subsequently investigated (Figure 3a). Comparably to the changes in H3K27me3 levels (Figure 2b,c), HMT activity was also found to be reduced upon the application of compressive forces in both the PA cultures and BSA control. Likewise, this reduction was significantly weaker in the PA-treated HPdLFs, which resulted in a higher HMT activity than in the dual-stressed BSA control.

To evaluate whether these somewhat small changes in H3K27 trimethylation and HMT activity were relevant for the pro-inflammatory effects of PA in dual-stimulated HPdLFs, we inhibited the core enzymes of the PRC2, EZH1 and EZH2 with UNC1999. This epigenetic inhibitor competes with the EZH1/EZH2 cofactor *S*-adenosyl methionine (SAM), thereby impeding proper histone lysine methylation [57].

To avoid potential toxicity, we first examined the metabolic activity of HPdLFs treated for six hours with different concentrations of UNC1999 (Figure 3b). This showed a slight positive effect of UNC1999, with the strongest effect occurring at a concentration of 1.00 µM. The activity of H3K27-related histone methyltransferases (HMTs) was reduced to 23.23% ± 2.56 in HPdLFs treated for six hours with 1.00 µM UNC1999, though lower inhibitor concentrations also resulted in a robust inhibition of HMT activity (Figure 3c). However, for further analysis, we decided to use 1.00 µM UNC1999, which was within the typical range used in previously published in vitro studies [58,59].

While comparable stimulations with compressive force and *P. gingivalis* LPS did not excessively affect cell survival [60,61,62], PA cultures showed slightly increased cell death rates [42]. To exclude the possibility that the combination of all the stress factors and UNC1999 induced cytotoxic effects, we examined the metabolic activity of those cells (Figure 3d). The analysis revealed a slight reduction by 5.8% ± 0.8 upon PA exposure in DMSO controls, which was not changed by additional UNC1999 treatment (5.9% ± 0.3; *p*-value, 0.9967). It should be noted that the metabolic activity of cells treated with UNC1999 was significantly higher than that of the corresponding DMSO controls in both BSA and PA cultures, confirming the previous results shown in Figure 2b. Furthermore, to rule out decreased cell survival due to the combination of all the conditions, we examined the number of apoptotic cells using TUNEL assays (Figure 3e,f). Consistent with previous analyses [42], PA cultures showed slightly increased rates of cell death in both the DMSO control and after UNC1999 treatment. However, these were not significantly different from each other (*p*-value, 0.9657).

To validate the inhibitory effect of 1.00 µM UNC1999 on H3K27 trimethylation in dual-stressed PA cultures, we analyzed HMT activity (Figure 3g). In both BSA control and PA cultures, UNC1999 treatment resulted in a decrease in HMT activity compared to the DMSO controls, confirming its inhibitory effect in the experimental setting. Thereby, PA-treated HPdLFs then showed comparable activity for the H3K27-specific HMT compared to the BSA control.

To illustrate the inflammatory processes, we performed an adhesion assay with monocytic THP1 cells (Figure 3h,i). Non-adherent THP1 cells recognize pro-inflammatory cytokines in the medium supernatant of stressed HPdLFs and attach themselves to appropriate sites in response to these signals. Therefore, the extent of a pro-inflammatory response can be assessed by evaluating the number of adherent CMFDA-stained THP cells relative to the HPdLF cell number. Comparably to previous results [39], PA exposure led to an enhanced inflammatory response of dual-stimulated HPdLFs compared to BSA controls, even in the presence of DMSO. While the by UNC1999-mediated reduction in the activity of EZH1 and EZH2 did not alter the number of adherent THP1 cells in the dual-stimulated BSA control, it reduced the excessive activation of monocytic cells in the PA cultures to a level comparable to that in the BSA controls.

To further investigate the effects of UNC1999 on inflammatory processes, we determined the expression of *COX2* (Figure 3j), a gene encoding an important cytokine that appears to be relevant to the PA-induced excessive inflammatory response of dual-stimulated HPdLFs [39]. Under the DMSO control condition, the *COX2* expression was significantly higher in PA cultures than in BSA controls. This upregulated *COX2* transcription was inhibited by the application of UNC1999. To confirm the potential impact of altered *COX2* levels on the secretion of related cytokines, we analyzed the secreted PGE2 levels in the supernatant of dual-stimulated HPdLFs that were additionally treated with UNC1999 (Figure 3k). The results supported the previously suggested assumption in the context of *COX2* expression, as the excessive secretion of PGE2 in dual-stimulated PA cultures was attenuated by UNC1999.

Together, these data suggest that changes in the reduction of H3K27me3 in dual-stimulated PA cultures via altered HMT activity may contribute to an excessive inflammatory response by HPdLFs, possibly via COX2/PGE2 regulation.

### 3.4. Palmitic Acid Causes Enhanced Trimethylation of H3K27 at Il10 Promoter-Associated Regions in Dual-Stimulated HPdLFs

**Figure 4:**
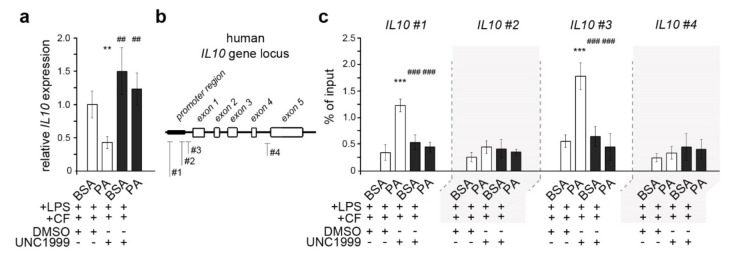
Palmitic acid exposure of dual-stressed HPdLFs resulted in decreased H3K27 trimethylation near *IL10* promoter regions associated with decreased *IL10* expression. (**a**) Quantitative expression analysis of *IL10* in human periodontal ligament fibroblasts (HPdLFs) exposed to palmitic acid (PA) and simultaneously stimulated with compressive force (CF) and *P. gingivalis* LPS after treatment with 1.00 µM UNC1999 compared to DMSO-treated BSA+LPS+CF control. (**b**) DNA primer locations in the *IL10* gene locus indicating promoter-associated pairs (#1, #2 and #3) and a non-promoter-associated pair (#4). (**c**) Quantitative analysis of the associations of H3K27me3 with specific *IL10* gene regions shown in (**b**) for dual-stimulated HPdLFs exposed to PA after UNC1999 treatment in comparison to the respective controls. Data were normalized to the sample input and IgG controls. **/## *p* < 0.01; ***/### *p* < 0.001; **/*** in relation to BSA+LPS+CF+DMSO; ##/### in relation to PA+LPS+CF+DMSO. One-way ANOVA and post hoc test (Tukey) were used for analysis.

H3K27me3 represses nearby genes, and a COX2-associated repressor appeared to be increasingly repressed via the epigenetic modification in dual-stimulated PA cultures. In this context, we next investigated the expression of the gene encoding the anti-inflammatory cytokine interleukin 10 (IL-10; Figure 4a), which has been shown to regulate *COX2* transcription [63] and whose expression can be affected by fatty acid-dependent changes in histone modifications [48]. We detected a significantly lower *IL10* transcription in dual-stimulated PA cultures compared to BSA controls, supporting our hypothesis.

To validate the impact of repressive H3K27 trimethylation on *IL10* gene activity, we performed the chromatin immunoprecipitation of DNA segments bound to H3K27me3. Further analysis of the association of trimethylated H3K27 at *IL10* promoter regions by quantitative PCR on H3K27me3-bound DNA revealed increased levels of this repressive histone mark at two *IL10* promoter-associated sides (#1 and #3) in dual-stressed HPdLFs exposed to PA (Figure 4b, white bars in Figure 4c). The non-promoter region (#4) was used as an internal control and showed no changes in H3K27me3 association; one promoter-associated position behaved similarly (#2). The enhanced association of H3K27me3 with *IL10* promoter sites (#1 and #3) was equalized by the inhibition of EZH1 and EHZ2 using UNC1999, the results appearing similar to those for the BSA controls (Figure 4b, black bars in Figure 4c). In conclusion, enhanced association with trimethylated H3K27 may be the underlying cause of the decreased expression of *IL10* in dual-stressed HPdLFs treated with PA, which, in turn, may cause enhanced COX2/PGE signaling under these conditions.

## 4. Discussion

This study investigated the impact of palmitic acid-induced epigenetic changes on the hyperinflammatory responses of HPdLFs that were simultaneously stimulated with *P. gingivalis* LPS and a compressive force. While the force-induced increase in global H3K acetylation was not affected by PA in LPS-stimulated HPdLFs, the reduction in global H3K27 trimethylation was less pronounced in PA cultures. Inhibitor studies have suggested that enhanced EZH1/EZH2 activity in dual-stimulated PA-exposed HPdLFs is a cause for enhanced COX2/PGE2 signaling. Since EZH1/EZH2-modulated H3K27me3 is a repressive mark, increased association with the *IL10* promoter provides a possible molecular mechanism via diminished IL-10-associated *COX2* downregulation for excessive inflammation in dual-stimulated PA cultures.

Epigenetic alterations to pro-inflammatory cytokine genes are well described for both periodontitis and obesity-associated hyperlipidemia [5,64]. Furthermore, mechanical forces were reported to alter histone marks, thereby mediating force-dependent changes in gene expression [48,65]. It is, therefore, likely that epigenetic transcriptional control represents a common level at which mechanical and bacterial influences, and obesity-related hyperlipidemic effects, may interfere.

We found force-induced changes in global H3K acetylation and H3K27 trimethylation in LPS-stimulated HPdLFs, which, however, were only different for repressive H3K27me3 under PA exposure. This is surprising, since in compressed HPdLFs cultured with monounsaturated oleic acid, an increase in global histone acetylation has been detected [48]. However, in this recent study, fibroblasts were not additionally stimulated using *P. gingivalis* LPS, which has been shown to increase HAT expression in human periodontal ligament stem cells (HPdLSCs) [66] and, therefore, any minor palmitic acid-induced changes in histone acetylation processes may have been overridden. This might have been due to the different pathways that convert lipids to acetyl-CoA, which is necessary for histone acetylation [21,35]. While long-chain fatty acids such as PA need to be actively transported across the mitochondrial membrane, where acetyl-CoA conversion takes place [67], shorter fatty acids, including pathogenic lipid metabolites, could diffuse across those membranes, leading to rapid oxidation to acetyl-CoA [68]. However, it is also known that different fatty acids can have varying influences on histone acylation by regulating distinct enzymes [69].

In accordance with other studies [54,70], we detected reduced global H3K27 trimethylation in HPdLFs that were challenged by *P. gingivalis* LPS and a compressive force. However, at least at the RNA level, we could not detect the altered expression of the major H3K27-HMT EZH1 and EZH2, which other studies have shown in force-stressed HPdLSCs and pathogenically stimulated human B cell lymphoma cells, such as BCBL1 [54,70]. However, we did not analyze the protein levels, which do not necessarily correlate with RNA expression [53]. This may be one reason why we detected increased global H3K27me3 upon PA exposure in dual-stressed HPdLFs, although gene expression levels were not altered. In this context, post-translational protein modifications, such as acetylation, methylation, phosphorylation, ubiquitination and O-GlcNAcylation, can also regulate protein function and be influenced by various environmental factors [71]. They were shown to affect the stability and nuclear localization of components of PRC2, its HMT activity and the binding of other proteins [56]. In this regard, we could show altered H3K27-specific HMT activity in dual-stimulated HPdLFs that were exposed to palmitate. However, this does not necessarily exclude the possibility that other HMTs may also contribute to the PA-associated changes. Moreover, it should be mentioned that the PA-related increases in H3K27me3 and HMT activity contradict studies reporting reduced levels of this histone mark [37] or the enhanced expression of associated histone demethylases (HMDs), such as JMJD3 [72], upon stimulation with this fatty acid. There are a variety of possible reasons for these contradictions, including the different cell types used, such as human urine-derived podocyte-like epithelial cells [37] and human and murine monocyte macrophages [72]. In addition, different PA concentrations and the combination of several stimuli possibly led to different changes in HMT or HMD activity.

Previously, we reported that PA exposure excessively promoted COX2/PGE2 signaling in *P. gingivalis* LPS-stimulated HPdLFs that were simultaneously stressed for six hours by compressive forces, resulting in the enhanced activation of monocytic THP1 cells [39]. As with our previous study, we detected increased PGE2 secretion in dual-stimulated HPdLFs, even when they were additionally cultured with DMSO as an inhibitor control. This is consistent with the literature describing increased PGE2 levels in various cells upon both compression and *P. gingivalis* stimulation in addition to fatty acid exposure [16,73,74,75,76,77,78]. In this study, we observed a proportionally increased expression of *COX2* in dual-stressed HPdLFs after PA exposure. This should be interpreted with caution, as it could also have been triggered by DMSO eliciting pro- and anti-inflammatory effects in a manner dependent on the cell type, concentration and duration [79]. However, both the excessive *COX2* expression and PGE2 secretion induced by PA exposure in dual-stimulated HPdLFs were counterbalanced by UNC1999, which reduces H3K27 trimethylation [57]. The transcriptional repression of *COX2* is related to high levels of H3K27me3 associated with the gene promoter region; when this mark was removed by EZH2-specific inhibition with DZNep, increased expression was observed [80]. However, other epigenetic modifications, such as histone acetylation and DNA methylation, modulating *COX2* transcription highlight a complex regulatory network for this important mediator of inflammatory signaling [80,81]. Moreover, we used UNC1999, which also inhibits EZH1 in a concentration-dependent manner [57]. Since EZH1 has similar functions but no fully overlapping targets with EZH2, potential differences could arise from the additional inhibition of EZH1. This is also supported by the study of Yamagishi et al., who reported different clusters of regulated genes for both HMTs and a more important role of EZH1 in the inflammatory response [82]. This may also explain why UNC1999 attenuated the excessive activation of THP1 cells to control levels in dual-stimulated HPdLFs exposed to PA. Future studies may elucidate the specific roles of both H3K27-HMTs in this complex regulation of the COX2/PGE2-driven inflammatory response.

Our study provides evidence that epigenetically modulated *IL10*-related *COX2* regulation is altered in PA-exposed HPdLFs that are concomitantly stressed by mechanical and bacterial stimuli, potentially resulting in a hyperinflammatory response. The negative regulatory effect of the anti-inflammatory cytokine IL-10 on *COX2* expression has been described comprehensively [63]. Similarly, the palmitic acid stimulation of adipocytes resulted in diminished *IL10* expression associated with reduced protein secretion [83], further promoting the pro-inflammatory effect of this fatty acid. We detected enhanced levels of H3K27 trimethylation at two positions in or close to the *IL10* promoter region of dual-stressed HPdLFs exposed to PA. The transcriptional repression of *IL10* by H3K27 trimethylation is also reported for other cell types, including T helper cells (Th)0, Th2 and iTreg cells [84] and Th17 cells [85]. In this regard, EZH2 knockdown resulted in increased levels of IL-10 [84], which we also observed in terms of gene expression due to pharmacological inhibition with UNC1999. On the basis of our data, it can be speculated that IL-10 is inhibited by a PA-mediated increase in H3K27me3 and, therefore, may fail to limit the COX2/PGE2-driven inflammatory response in dual-stimulated HPdLFs. In addition, one could speculate that H3K27 trimethylation is one general mechanism for *IL10* transcriptional repression, since it is barely detectable in the HPdLFs that were not stressed by compressive forces (data not shown). However, due to the high complexity of the epigenetic profile and its dependence on environmental influences, along with the complex dynamics of histone modifications [86], this could only be elucidated by additional gene-specific analyses.

Our study has several limitations, including the use of a single fatty acid and LPS as our only pathogenic stimuli, each at one specific concentration, and the study of a single duration of compressive force. Even though the ratio of palmitic acid to BSA has been reported for the serum levels of obese patients, in vivo, the concentrations of other fatty acids are also decisive for hyperlipidemia-related health problems [87,88,89,90,91]. This makes further clinical studies indispensable. After six hours of the application of the compressive force, one could detect an early phase of the inflammatory response; however, COX2/PGE2 signaling was reported to already be increased [92,93]. More critical is the remarkable complexity of epigenetic regulation. In this context, changes in one modification require adaptations of other modifications. It has been shown that a reduction in H3K27 trimethylation can lead to increased global and gene-specific levels of activating modifications, such as H3K4me3 and H3Kac [86]. However, elucidating these changes in detail was beyond the scope of this study, which aimed to establish an initial potential link between the hyperinflammatory response of dual-stressed hyperlipidemic PdL fibroblasts and the epigenetic regulation of potential key factors.

Due to the potential reversibility of this hyperlipidemia-related adaptation of the inflammatory response, H3K27me3 inhibitors may be of therapeutic interest in addition to the HDAC inhibitors that have already been explored [94]. This is of particular interest considering the increase in obesity-associated hyperlipidemia patients requiring orthodontic treatment and the relevance of periodontal disease in light of the associated risks, such as tooth root resorption and tooth loss. In the treatment of periodontitis, there are already therapeutic approaches involving the modulation of the inflammatory responses of the host cells [95]. Epigenetic remodeling may provide a novel platform with which to overcome unfavorable disease-induced cellular changes by treatment with epigenetic inhibitors during tooth movement.

## 5. Conclusions

Highly dynamic epigenetic modifications of histones can modulate gene expression in response to a wide variety of environmental cues. Our study illustrated, for the first time, that obesity-associated hyperlipidemic states can influence epigenetic changes initiated in bacterially stimulated fibroblasts via mechanical forces affecting their inflammatory responses. Thus, the palmitic acid-associated hyperinflammatory response to the cellular compression of *P. gingivalis* LPS-stimulated human PdLFs seems to be realized through the downregulation of the COX2 repressor IL-10 through enhanced H3K27 trimethylation close to its gene promoter. The obtained results strongly suggest a central role of metabolism-associated changes in the epigenetic regulation of soft and hard tissue remodeling due to pathogenic and mechanical stress, offering promising potential for future targeted therapy.

## Figures and Tables

**Table 1:** qPCR primer sequences of human genes indicated in the 5′-3′ direction. bp, base pairs; fw, forward; Length, amplicon length; PRC, polycomb repressive complex; rev, reverse.

Gene	Gene Symbol	NCBI Gene ID	Primer Sequence	Length
CREB-binding protein	*CREBBP*	1387	fw: CCAAGACCTGCGATTTCCAC rev: TTTTGATGTCCCAGAAGCGG	100 bp
Embryonic ectoderm development	*EED*	8726	fw: TGCGATGGTTAGGCGATTTG rev: CCAAATGTCACACTGGCTGT	158 bp
E1A-binding protein p300	*EP300*	2033	fw: TGACCAAGGGAGACAGCAAA rev: GAGGCGGATCACAAAGAAGAC	182 bp
Enhancer of zeste 1 PRC2 subunit	*EZH1*	2145	fw: CGAGAATGTGACCCTGACCT rev: TTATGAAGGTGCCCCATCCG	154 bp
Enhancer of zeste 2 PRC2 subunit	*EZH2*	2146	fw: ACAGTTCGTGCCCTTGTGTG rev: CACTCTCGGACAGCCAGGTA	148 bp
Histone deacetylase 1	*HDAC1*	3065	fw: AGCTCCACATCAGTCCTTCCA rev: TTCGTCCTCATCGCCACTCT	170 bp
Histone deacetylase 2	*HDAC2*	3066	fw: ACTGATGCTTGGAGGAGGTG rev: CTGGAGTGTTCTGGTTTGTCA	185 bp
Histone deacetylase 3	*HDAC3*	8841	fw: GCTGGGTGGTGGTGGTTATA rev: TTCTGATTCTCGATGCGGGT	174 bp
Interleukin 10	*IL10*	3586	fw: AGCCATGAGTGAGTTTGACA rev: AGAGCCCCAGATCCGATTTT	141 bp
Lysine acetyltransferase 8	*KAT8*	84148	fw: GCAAGATCACTCGCAACCAA rev: AGTCTTCGGGGAATGGTGAG	195 bp
Nuclear receptor coactivator 3	*NCOA3*	8202	fw: GGCTCTATTCCCACATTGCC rev: CCCAGTTGGTTAGATGCTGC	158 bp
Prostaglandin endoperoxide synthase 2	*PTGS2* *(COX2)*	5743	fw: GATGATTGCCCGACTCCCTT rev: GGCCCTCGCTTATGATCTGT	185 bp
Ribosomal protein L22	*RPL22*	6146	fw: TGATTGCACCCACCCTGTAG rev: GGTTCCCAGCTTTTCCGTTC	98 bp
SIN3 transcription regulator family member A	*SIN3A*	25942	fw: GAGCAGCAGGAAAAGGAAGG rev: TGTAGACGCTTGCTTACACG	200 bp
SUZ12 polycomb repressive complex 2 subunit	*SUZ12*	23512	fw: CGGACCAGTTAAGAGAACACC rev TTGTGGACGGAGAGGTAAGC	181 bp
TATA box-binding protein	*TBP*	6908	fw: CGGCTGTTTAACTTCGCTTCC rev: TGGGTTATCTTCACACGCCAAG	86 bp

**Table 2:** qPCR primer pairs located in the human *IL10* in promoter regions (#1, #2 and #3) and a non-promoter region (#4) indicated in the 5′-3′ direction. bp, base pairs; fw, forward; Length, amplicon length; rev, reverse.

Location	Label	Primer Sequence	Length
Promoter region	#1	fw: TGAAGAAGTCCTGATGTCAC rev: TTACCTATCCCTACTTCCCC	187 bp
Promoter region	#2	fw: AGCACTACCTGACTAGCATA rev: AGAGACTGGCTTCCTACAG	192 bp
Promoter region	#3	fw: GGGGACCCAATTATTTCTCA rev: TGGGCTACCTCTCTTAGAAT	188 bp
Non-promoter region	#4	fw: GCTTAGAGCGTTTCCAGACC rev: CTCCCCACTGTAGACATCCA	131 bp

## Data Availability

The datasets of this study and the data of further controls not included here are available upon reasonable request from the corresponding author. The microscopic data are not publicly available due to the very large size of the microscopy images.
